# Raising awareness about the unintended consequences of hand sanitiser in children

**DOI:** 10.4102/safp.v63i1.5278

**Published:** 2021-06-28

**Authors:** Olive Khaliq, Princess Z. Mkhize, Jagidesa M. Moodley

**Affiliations:** 1 Department of Obstetrics and Gynecology, Women’s Health and HIV Research Group, Nelson Mandela School of Medicine, Faculty of Clinical Medicine, University of KwaZulu-Natal, Durban, South Africa

**Keywords:** alcohol-based sanitiser, COVID-19, ocular injury, skin irritations, children

## Abstract

The use of hand sanitisers is common practice to prevent the spread of coronavirus disease 2019 (COVID-19). However, the safety thereof requires consideration as this may be hazardous in children. Recent studies have shown that the misuse and increased unsupervised availability of alcohol-based hand sanitisers may result in adverse events in children such as skin irritation, dryness, cracking and peeling. Unintentional or intentional ingestion of hand sanitisers in children under the age of 12 years may occur because of the colour, smell and flavour added to it. Consumption of alcohol in children may result in hypoglycaemia, apnoea and acidosis. This allows the invasion of other bacterial and viral infections. Children may also rub their eyes with sanitised hands and cause ocular injury. Therefore, the use of hand sanitisers in general needs to be revised in both children and adults. Other interventions on lowering the risk of adverse events because of misuse of hand sanitiser should be practised more often. These include promoting washing of hands over sanitisers where possible, training children on how to use hand sanitisers and creating awareness of the dangers if ingested or in contact with the eyes.

## Background

Contagious diseases utilise the skin (hands), nostrils (inhalation), eyes (contact) and mouth (ingestion) as the port of entry for infection.^1^ Chemicals such as hand sanitisers have been formulated and designed to kill dermal microbial and viral infections.^1^ Prior to the coronavirus disease 2019 (COVID-19) pandemic, the use of hand sanitisers was encouraged mainly in public spaces and hospital wards. However, after the pandemic, hand sanitisers have become the “new norm” particularly if hand washing with soap and water is unavailable. Sanitisers are either water-based or alcohol-based. The most effective type is the alcohol-based sanitiser (60% – 95% alcohol content).^1^

The first cases of coronavirus were reported at the end of December 2019. The World Health Organization (WHO) declared the coronavirus pandemic only in March 2020.^1^ To reduce and prevent individual spread and spread from contact with surfaces that have been contaminated, wearing of masks, social distancing and frequent sanitising with alcohol-based sanitiser or hand washing with soap and water have been recommended in a large number of countries.^2^ These measures have decreased the spread of the virus substantially.^2^ However, the frequent use of alcohol-based sanitiser may be hazardous to the skin, eyes and chest if inhaled, especially in small children because they have very sensitive skin. Therefore, young children in particular should be trained to avoid frequently touching their faces and sucking their hands.^3^

This report focuses on the unintended consequences of the frequent use of hand sanitisers in children. The effects of the contents and the risk thereof to small children aged 12 and below will be highlighted. Small children have little understanding of the dangers of their surroundings and of the dangers of COVID-19. They are encouraged to wear masks, practice social distancing and to sanitise their hands regularly, but may not be encouraged enough to avoid touching their faces or mouths with sanitised hands and this may create health complications. Furthermore, toddlers and those under the age of 6 years may find the scented sanitisers appealing and may ingest them directly or indirectly through their hands after sanitising.^4^ In addition, recent reports showed that ocular injuries occur because of inadvertent exposure to contents of hand sanitisers from those installed at waist-level height of an adult but at eye level or above for a child causing induced ocular injury (IOI) through direct exposure.^4^

## The formulation of hand sanitisers

Alcohol-based sanitisers are composed of ethanol or isopropyl, distilled water, octenidine dihydrochloride and phenoxyethanol.^5^ Some sanitisers may be composed slightly differently with ethanol and isopropyl and aminomethyl propanol.^6^ The concentrations of ethanol vary with different companies. Some may contain 60% – 95% alcohol, whilst many contain 70%.^6^

As a result of the coronavirus pandemic, the WHO has enforced changes to the concentrations of ethanol and isopropyl in hand sanitisers. The recommended concentration has been increased to 96% of ethanol and 99.8% of isopropyl at a lower volume.^7^ Therefore, the standardised composition of hand sanitisers that are safe for home use are as follows: ethanol (80%) volume/volume (v/v), hydrogen peroxide (0.125%) v/v and glycerol (1.45%) v/v for formulation A and isopropyl alcohol (75%) v/v, hydrogen peroxide (0.125%) v/v and glycerol (1.45%) v/v for formulation B.^6^ The WHO sanitiser formulations have been in practice in healthcare settings during viral outbreaks.^8^

## The toxicity of hand sanitiser

These reagents are 100% effective in killing the coronavirus but are toxic and may lead to serious complications and sometimes death if ingested.^3^ The regular use of ethanol-based sanitisers has shown to be harsh on the skin, resulting in skin irritation on the hands.^6^ However, Kramer et al. reported that the regular use of ethanol has no side effects on the skin and that it was safe to use in adults, and this was based on their findings that showed that the concentration of alcohol absorbed through skin is less than the toxic level for humans.^9^

Oral ingestion of alcohol-based products such as mouthwash, cosmetics and hand sanitiser become hazardous at a certain concentration level. An oral consumption of > 400 mL/dL contains 80% ethanol and this may be toxic and fatal.^10,11^ Symptoms associated with high concentrations of alcohol include nausea, vomiting, epigastric pain and central nervous system disorders.^10^ Concentrations above 300 mL/dL may compromise the body and result in complications, which include respiratory depression, and this may cause conditions such as respiratory arrest hypothermia, cardiac dysrhythmias, cardiac arrest, hypoglycaemia, ketoacidosis and hypotension.^12^

The ingestion of alcohol in children is of greater danger than it is in adults. Some hand sanitisers have appealing scents that may appetise children resulting in ingestion of the substance. This may cause hypoglycaemia because their liver has limited glycogen stores.^13,14,15^ Furthermore, other researchers have reported different complications associated with the ingestion of ethanol in children, namely apnoea, acidosis and sometimes coma.^16,17,18^ Contact of the skin may also lead to allergic reactions that cause skin irritation and eye infections because children tend to touch or rub their eyes frequently.^3^ Children under the age of six are reported to unintentionally ingest sanitiser and touch their eyes whilst those over the age of six years intentionally ingest it because of temptations stirred up by the smell and colour of the sanitiser.^3^

## The risk of other infections because of the excessive application of hand sanitiser

The excessive use of hand sanitiser irritates the hands because ethanol enhances penetration of the skin and results in thinning of the epidermis, dryness, cracking and subsequent peeling.^6^ Thinning of the skin decreases protection from viral invasion and peeling exposes the dermis to other microbes and viruses.^6^ Vogel et al. reported that the excessive use of sanitiser increases the risk of norovirus, which is highly infectious and may result in severe cases of vomiting and diarrhoea.^19^ Children have very sensitive skin; therefore, the ethanol in hand saniters penetrate the skin easily, resulting in higher susceptibility to adverse events than in adults.

The United States Centre for Disease and Control (CDC) has proposed the use of alcohol-based hand rubs (ABHRs) and hand washing to fight the COVID-19 pandemic. This is because of the structural characteristics of coronaviruses, which are enveloped approximately by 109 viruses with lipid bilayer and are easily inactivated by alcohol.^20^ The guidelines suggest the use of ABHRs over hand washing where the hands appear clean to ensure greater compliance. However, the American Academy of Paediatrics has warned against the possible toxicity of ABHRs containing isopropanol on children, if accidentally consumed. Guidelines have been issued to add bitter tasting compounds to ABHRs to discourage its consumption by children. Still, there is a need for vigorous awareness campaign to educate the public about potential consequences of its use. Parents need to ensure that they read all labels before applying such products on children. Caution needs to be applied whilst giving complete control of these preparations to children.^20^ Appropriate guidelines need to be followed regarding the usage of sanitisers by children such as putting travel packs and pocket-sized bottles in their backpacks.^20^

Mahmood et al. reported that the American Association of Poison Control Centre (AAPCC) 9504 alcohol-based hand sanitiser exposure cases in children under the age of 12 years in the first five months of the pandemic in 2020. They stated that even a small amount of alcohol can cause alcohol poisoning in children and this was accountable for confusion, vomiting and drowsiness and in severe cases, respiratory arrest and death.^6^ Mahmood et al. mentioned that the AAPCC, 2020 recognised that the quantity of hand sanitisers should be considered before they can be supplied.^6^

The following measures can be taken to prevent inadvertent harmful effects of hand sanitisers:

- Promotion of frequent hand washing with soap and water over hand sanitiser in children and having handwashing facilities readily accessible to children should be considered
- education and training of children on how to use hand sanitiser
- recommendations to Departments of Education and public health providers to put caution signs next to sanitiser dispensers and to install eye washing devices to immediately deal with an inadvertent eye touching or accidental eye spraying by children
- Parents and the general public should be provided with information regarding safety measures specific for children on the dangers of hand sanitisers.

## Conclusion

The inadvertent exposure of hand sanitiser to children poses a high risk of potentially life-threatening complications. Furthermore, the misuse and increased unsupervised availability of alcohol-based hand sanitisers may result in adverse events in children. The frequent hand washing with soap and water should be encouraged as a common practice for children.
